# Skeletal muscle laminin and MDC1A: pathogenesis and treatment strategies

**DOI:** 10.1186/2044-5040-1-9

**Published:** 2011-03-01

**Authors:** Kinga I Gawlik, Madeleine Durbeej

**Affiliations:** 1 Muscle Biology Unit, Department of Experimental Medical Science, Lund University, 221 84 Lund, Sweden

## Abstract

Laminin-211 is a cell-adhesion molecule that is strongly expressed in the basement membrane of skeletal muscle. By binding to the cell surface receptors dystroglycan and integrin α7β1, laminin-211 is believed to protect the muscle fiber from damage under the constant stress of contractions, and to influence signal transmission events. The importance of laminin-211 in skeletal muscle is evident from merosin-deficient congenital muscular dystrophy type 1A (MDC1A), in which absence of the α2 chain of laminin-211 leads to skeletal muscle dysfunction. MDC1A is the commonest form of congenital muscular dystrophy in the European population. Severe hypotonia, progressive muscle weakness and wasting, joint contractures and consequent impeded motion characterize this incurable disorder, which causes great difficulty in daily life and often leads to premature death. Mice with laminin α2 chain deficiency have analogous phenotypes, and are reliable models for studies of disease mechanisms and potential therapeutic approaches. In this review, we introduce laminin-211 and describe its structure, expression pattern in developing and adult muscle and its receptor interactions. We will also discuss the molecular pathogenesis of MDC1A and advances toward the development of treatment.

## Introduction

**Figure 1:**
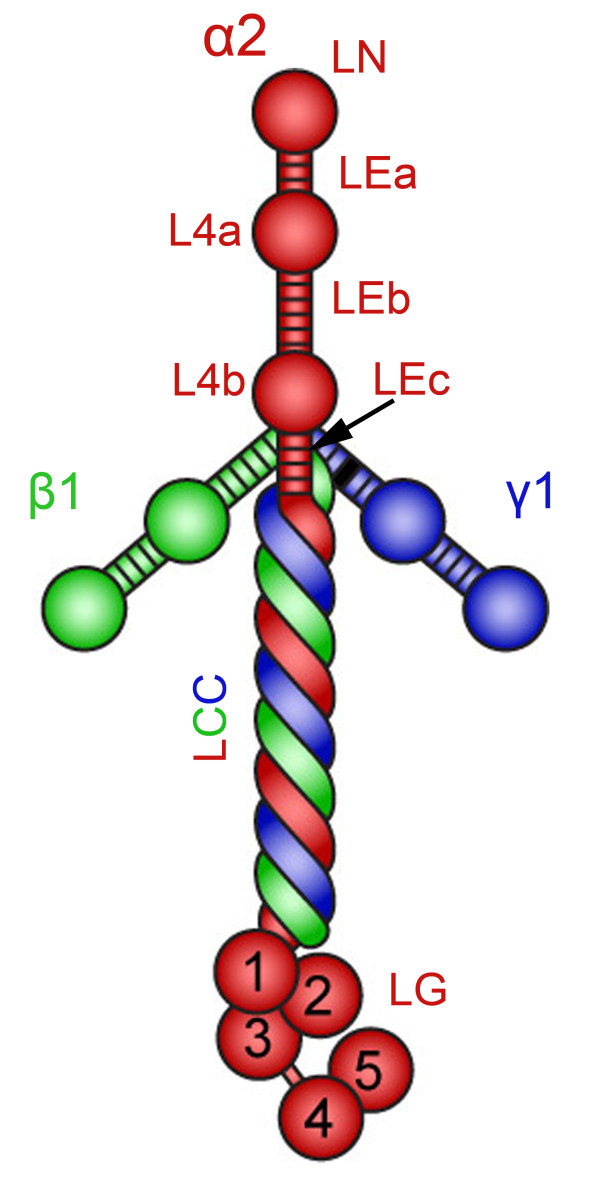
**Scheme of laminin-211 heterotrimeric structure**. Laminin α2 chain is depicted in red, β1 in green and γ1 in blue. Laminin α2 chain consists of: the N terminal globular domain (LN); tandem rod domains of epidermal growth factor (LEa, LEb, LEc), separating the LN, L4a and L4b globular domains; the laminin coiled-coil (LCC) domain that tangles with the LCC domains of the β1 and γ1 chains; and the C-terminal laminin globular (LG) domains.

The basement membrane is a thin scaffold of specific extracellular protein networks associated with various cell types, including muscle fibers. This specialized framework of extracellular matrix (ECM) provides important functional cues to cells. Laminins comprise a family of glycoproteins that are major components of all basement membranes [1]. Occurrence of a laminin molecule in hydra, one of the oldest multicellular organisms, indicates that laminins existed already 600 million years ago [2]. Laminins are large (400-900 kDa) heterotrimeric molecules composed of one α, one β and one γ subunit in a cruciform or T-shaped appearance. To date, five α, three β and three γ chains have been characterized. They represent the products of distinct genes that evolved by duplication and recombination of ancestral α, β and γ genes, hence they share sequence similarity. Currently, the trimers are named according to the composition of the α, β and γ chains and more than 15 different laminin isoforms, with various arrangements of laminin subunits, have been identified [3-5]. The first laminin isoform, laminin-111, was discovered more than 30 years ago in the Engelbreth-Holm-Swarm tumor [6]. Subsequently, laminin-211 (composed of α2, β1 and γ1 chains) (Figure 1) was isolated from placenta and was originally called merosin [7]. It is now well established that laminin-211 is the main laminin isoform in skeletal muscle [8,9], and identification of laminin α2 chain mutations in a severe form of congenital muscular dystrophy (merosin-deficient congenital muscular dystrophy; MDC1A) showed the importance of laminin-211 for normal muscle function [10].

### Laminin α2 chain gene and protein

The *LAMA2 *gene is located on chromosome 6q22-23 in humans and on chromosome 10 in mice [10-12]. The gene is composed of 65 exons that encode a protein with a predicted molecular mass of 390 kDa. However, it is cleaved by a furin-like convertase into a 300 kDa N-terminal segment and a 80 kDa C-terminal segment, which remain non-covalently associated [13-15]. Whether this proteolytic processing has functional consequences in muscle *in vivo *is not known. The laminin α2 chain has a similar domain organization to that of the other laminin chains, with several globular and rod-like regions. Domains LN, L4a and L4b form globular structures separated by rod-like spacers of LE domains (epidermal growth factor-like repeats), followed by a coiled-coil domain, and finally, the C-terminal end is composed of five homologous laminin globular (LG) domains (LG1 to LG5) (Figure 1) [3]. Key biologic activities have been mapped to several of these domains. The LN domain is essential for laminin polymerization into supramolecular networks and consequently for incorporation into basement membranes [16], and mutations in this domain reduce the ability of polymer formation [17]. The coiled-coil domain is involved in the formation of laminin heterotrimers, and the laminin α2 chain can assemble with the β1, γ1, β2 and γ3 chains to form laminins 211, 221 and 213 [5]. The laminin α2 LG domains at the C-terminus bind cellular receptors (dystroglycan and integrin α7β1) [15,18], and such interactions are required for adhesion, basement-membrane assembly and downstream signaling events [19,20].

### Laminin-211 and other laminins in developing muscle

Myogenesis is a complex multistep process, but it has been found that muscle morphogenesis is strongly guided by ECM cues [21]. There is robust evidence that laminins are important for synaptogenesis [22-26], but their precise function in myogenesis is still not known. Each immature murine somite is surrounded by a laminin-111-rich basement membrane [27]. While entering the myotome during somite differentiation, muscle progenitors begin to form the myotomal basement membrane that separates the myotome and sclerotome [28], and laminin-111 seems to be fundamental for the initiation of its assembly, at least in mice [29]. After the initial myogenic events, formation of primary and secondary myotubes takes place. Basement-membrane remodeling and differential expression of laminin subunits is tightly correlated with these events. During the first fusion events in mice at embryonic day (E)11, laminin-211 and laminin-511 are the major heterotrimers of the newly formed basement membrane (the laminin α1 chain is still present at E11.5, but it is largely restricted to the ends of myotubes) [9]. Just before the fusion of secondary myotubes (at E14), expression of the laminin α4 chain increases dramatically, and it is deposited throughout the secondary myotube basement membrane by E15 [9]. In developing human muscle, the laminin α2 chain is present from around the seventh week of gestation, reaching maximum expression levels at week 21 [30,31], and the laminin α4 chain is strongly expressed at week 16 [32]. Additionally, the laminin α5 subunit was shown to be a major laminin α chain during myogenesis in humans, whereas the laminin α1 subunit was detected only in the developing myotendinous junction (MTJ) [31,33]. It is noteworthy that the laminin composition is also modified during development of specialized muscle sites, such as the neuromuscular junction (NMJ) and the MTJ [9,31,34].

Further changes om the laminin array in muscle basement membrane occur perinatally both in human and mouse as myotubes mature into myofibers. The levels of laminin α4 and α5 subunits markedly decrease at birth and are not detectable at the sarcolemma by the end of the first postnatal week [9,32,35]. Thus, the laminin α2 subunit is the only laminin α chain expressed in the extrasynaptic basement membrane. Interestingly, *in vitro *studies with myogenic cell lines found that both the laminin α1 and α2 chains possess myogenic properties, performing both shared and specific tasks in myogenesis [36,37].

Although several laminins are expressed in a distinct manner during myogenesis, none of the laminin α chains seems to be essential for this event [23,38,39]. Myogenesis occurs normally in patients and mice lacking laminin α2 subunit [11,12,40-43], even though myofibers are smaller at birth in patients with MDC1A [40,41]. It is possible that laminin α4 and/or α5 could compensate for absence of the laminin α2 chain in developing muscle, and studies of muscles devoid of several α chains would be therefore be interesting. Laminins containing the α2 chain are instead crucial in adult muscle, and this topic will be discussed in more detail in later sections.

### Laminin-211 and other laminins in mature muscle

**Figure 2:**
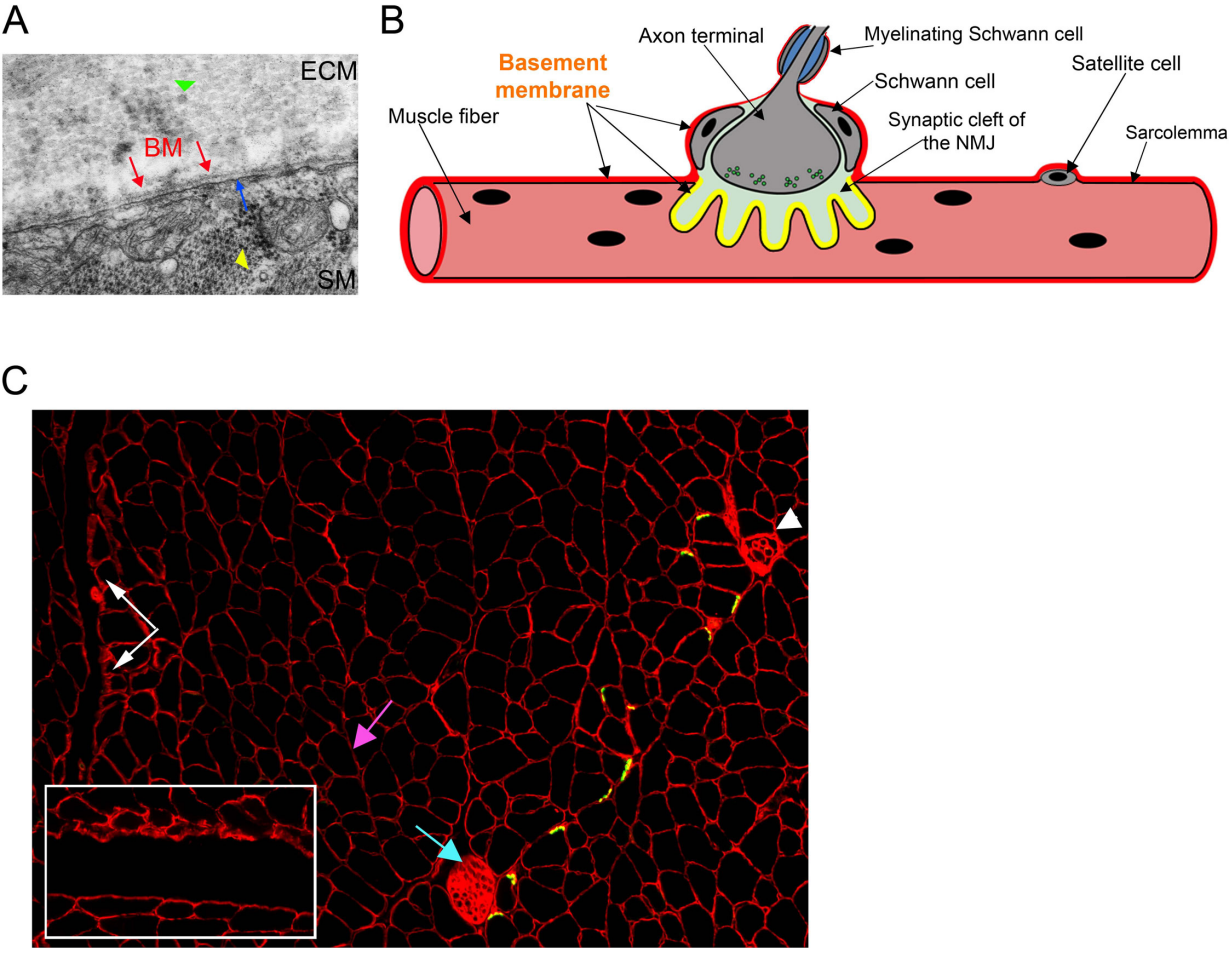
**Basement membrane (BMs) of the neuromuscular system**. **(A) **Electron microscopy of BMs in normal diaphragm muscle (SM, skeletal muscle). Red arrow = BM, blue arrow = sarcolemma, yellow arrowhead = sarcomere (cross-section), green arrowhead = collagen fibrils in the interstitial matrix (ECM). **(B) **Scheme of the motor innervation with distinct BMs. The BMs comprise a continuous layer around muscle fibers and Schwann cells of the peripheral nervous system (red layer), and there is a different composition of laminin chains and other extracellular matrix components at the neuromuscular junction (NMJ) (yellow layer). Muscle fibers and satellite cells are surrounded by an extrasynaptic BM containing mainly laminin-211. At the NMJ, laminins 221, 421 and 521 are the prominent laminin isoforms. Schwann cells at the NMJ and myelinating Schwann cells (blue = myelin) that frame the axon of the motor nerve are surrounded by a BM with slightly different laminin composition (laminins 211 and 411 as well as 221 and 421). **(C) **Laminin α2 chain expression in BMs of the neuromuscular system (immunofluorescent staining with the 4H8-2 antibody generated against the N-terminal domain of the laminin α2 chain). The laminin α2 subunit (red staining) forms the backbone of BMs that surround muscle fibers (extrasynaptic BMs) (purple arrow),underlie the NMJ (yellow) (the co-localization of laminin α2 chain (red staining) and acetylcholine receptors (green staining with α-bungarotoxin) is also shown), surround Schwann cells of the intramuscular peripheral nerve (blue arrow), surround muscle spindles (white arrowhead) and underlie the myotendinous junction (MTJ) (white arrows). Inset shows a higher magnification of the MTJ.

The basement membrane surrounding the mature muscle cell (Figure 2A,B) contains laminins 211 and 221 [8,9,44] (Figure 2C). To form a strongly crosslinked basement membrane, which provides significant structural support to muscle cells [45], laminins 211 and 221 bind to each other and to other matrix proteins including nidogens (which in turn (connect) the laminin network to the collagen network), fibulins and agrin [18,46]. There are also structurally and functionally specialized basement membranes within the skeletal muscle compartment (Figure 2B,C). The sites of muscle contact with motor nerves (the NMJ) encompass three basement membranes, each with a distinct laminin expression pattern; the extrasynaptic basement membrane, the basement membrane of the synaptic cleft, and the Schwann cell basement membrane (Figure 2B). The basement membrane within the synaptic cleft contains laminins 221, 421 and 521 at the sites of concentration of acetylcholine receptors (primary clefts), whereas the sites of concentration of sodium channels (folds of secondary clefts) include laminins 221 and 421 [9]. Basement membranes surrounding Schwann cells in the peripheral nervous system include mainly laminins 211, 411 [47] and probably 221 and 421 [48]. There is also a specialized junction where muscle abuts tendon (the MTJ) and laminins 211 and 221 are strongly expressed at this site [49,9] (Figure 2C).

Laminin-211 in the sarcolemmal basement membrane is extremely important for maintenance and stabilization of differentiated muscle [37,50], and absence of the laminin α2 chain leads to muscular dystrophy in humans and mice [10-12,40-43]. Subtle NMJ defects have also been reported in laminin α2 chain-deficient mice [51], but it is possible that these arise from muscle abnormalities caused by the dystrophic process. The laminin α4 and β2 chains, by contrast, have important roles in the NMJ. Mice devoid of the laminin α4 and β2 chainshave abnormal neuromuscular (synapses) [22,23,26], and laminin β2 chain deficiency in humans (Pierson syndrome) is characterized by muscular and neurologic defects in addition to kidney failure [52].

### Laminin receptors in skeletal muscle

**Figure 3:**
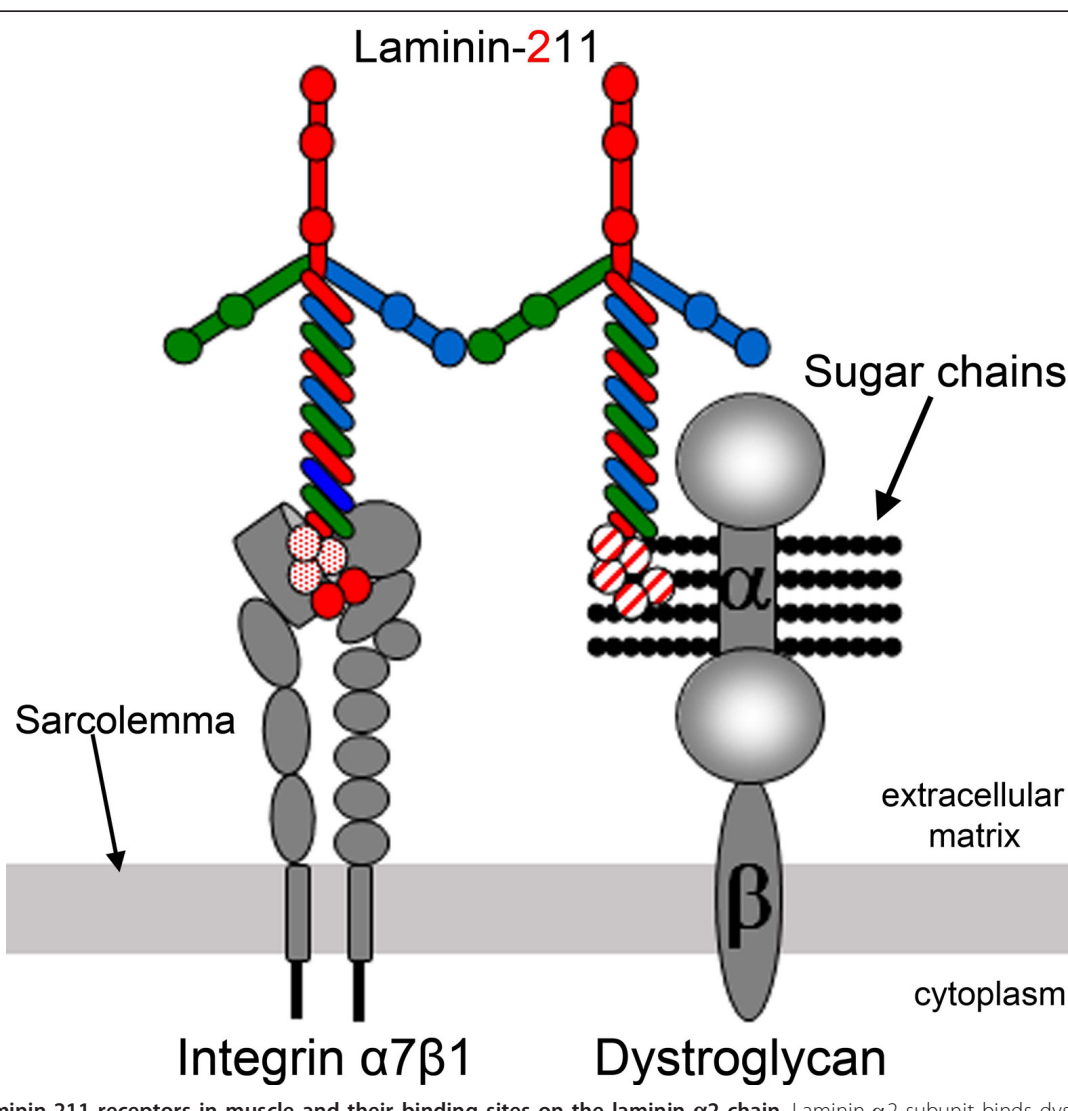
**Laminin-211 receptors in muscle and their binding sites on the laminin α2 chain**. Laminin α2 subunit binds dystroglycan and integrin α7β1 via the laminin globular (LG) domains. LG1 to 3 and 4 to 5 bind α-dystroglycan, whereas only LG1 to 3 binds integrin α7β1. Glycosylation of α-dystroglycan is important for laminin binding.

Transmembrane receptors that interact with laminin networks, connecting them to the cytoskeleton and intracellular signaling pathways, trigger the biologic functions of laminins. The two major laminin-211/221 receptors in skeletal muscle are dystroglycan and integrin α7β1 (Figure 3). Dystroglycan is a highly glycosylated, ubiquitously expressed protein that consists of two subunits, α-dystroglycan and β-dystroglycan [53]. In muscle, it forms the backbone of the multisubunit dystrophin-glycoprotein complex (DGC) linking laminin-211 to the intracellular components dystrophin and actin [54,55]. It was recently found that a phosphorylated *O*-mannosyl glycan on α-dystroglycan is required for laminin binding [56], which occurs through the LG domains of the laminin α2 chain [18]. Both laminin α2 LG1 to 3 and LG4 to 5 bind α-dystroglycan strongly (whereas individual modules do not bind, except for weak interactions with the LG3 domain) [14,18]. The crystal structure has been solved for the laminin α2 LG4 to 5 domain, and the α-dystroglycan binding site in the α2 LG4 to 5 domain has been defined [57]. Binding to α-dystroglycan is calcium-dependent [55]. The structure of the mouse laminin α2 LG4 to 5 domain showed that the two calcium ions, implicated in dystroglycan binding, are located in LG4 and LG5, respectively, and the extensive basic surface region between the calcium sites is proposed to bind α-dystroglycan [57].

Integrin α7β1 is the second transmembrane unit that links laminin-211 to the cytoskeleton [58-60] and binding occurs through the laminin α2 LG1 to3 domain with involvement of the coiled-coil domain [14,15]. However, the adaptor molecules that connect integrin α7β1 to the cytoskeleton remain to be identified [61], although talin [62] and integrin-linked kinase [63] are likely candidates. We also recently identified a novel integrin α7β1 interacting protein (Cib2), whose expression in muscle is dependent on the presence of the laminin α2 chain [64].

The significance of the laminin receptors for normal muscle function is emphasized by the fact that mutations in DGC components and post-translational defects in dystroglycan processing and mutations in the integrin α7 gene causes various forms of muscular dystrophy and myopathy [65,66]. Hence, there is strong evidence that both receptors contribute to linking laminin-211 to the cytoskeleton and mediate the effects of laminin-211 on muscle integrity and function. It has been shown that the two systems act synergistically [67,68], but separate roles have also been delineated [69,70]. Both dystroglycan and integrin α7β1 contribute to force production, but only dystroglycan is involved in anchoring the basement membrane to the sarcolemma [69]. Furthermore, different muscles may have different requirements for the laminin-dystroglycan interaction as it may not be crucial in diaphragm but important in limb muscle [70]. Nevertheless, many of the downstream events of the laminin-211-receptor interaction remain to be elucidated. Several signaling pathways may be affected, but the importance of each of those pathways in skeletal muscle is not obvious [71-76].

Finally, it should be noted that laminin-211 also binds other cell-surface receptors, although dystroglycan and integrin α7β1 may be considered as the major laminin-211 receptors in skeletal muscle. These other receptors include the syndecans and sulfated glycolipids [18,77]. Interestingly, sulfatides have been proposed to anchor laminin-211 by binding to its LG domains to initiate basement-membrane assembly and to engage the activation of receptors (dystroglycan and β1 integrins), at least in Schwann cells [20].

### Congenital muscular dystrophy type 1A

**Figure 4:**
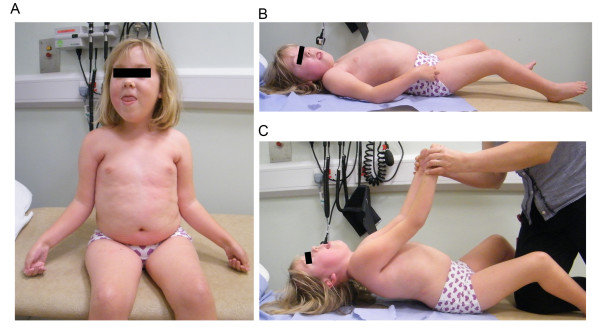
**Clinical features of a patient with merosin-deficient congenital muscular dystrophy (MDC1A) with complete laminin α2 chain deficiency**. **(A) **Facial weakness with an open mouth and reduced facial expression. Patient has developed bilateral elbow-flexion contractures, a fairly common sign in patients with MDC1A. **(B) **Bilateral knee-flexion contractures and lumbar hyperlordosis. **(C) **Truncal weakness and neck-flexion weakness (lack of head control) when the patient is pulled up from a lying position. Informed consent was obtained from the patient's parents for publication of the photographs.

Laminin α2 chain-deficient muscular dystrophy (MDC1A), showing autosomal recessive inheritance, was recognized as a particular form of congenital muscular dystrophy in 1994 when Tomé *et al*., found specific absence of the laminin α2 chain in patients [41]. Shortly after, the first causative mutations in the *LAMA2 *gene were identified, and a number of mutations have subsequently been reported [10,78,79]. It is now clear that complete laminin α2 chain deficiency leads to a severe phenotype, whereas partial deficiency may lead to a severe or a milder phenotype [80]. The estimated prevalence of congenital muscular dystrophy is around 7 × 10^-6 ^[81], and MDC1A accounts for approximately 40% of cases of congenital muscular dystrophy in Europe [78,79]. The clinical features of MDC1A include profound muscle hypotonia at birth and generalized muscle weakness accompanied by contractures that mostly affect the elbows, hips knees and ankles, along with scoliosis, kyphosis, increased creatine kinase levels, and delayed motor milestones (Figure 4). Patients may achieve unsupported sitting, but very few attain ambulation. Common serious complications of MDC1A include respiratory failure and feeding difficulties. Importantly, treatment with noninvasive ventilation and gastrostomy can greatly improve health. However, respiratory-tract infection is the commonest cause of death, which may occur in the first decade of life or anytime subsequently [78,79]. As the laminin α2 chain is also expressed in the central nervous system (CNS), peripheral nervous system and heart [8], these tissues are also affected to various degrees in MDC1A. Most patients (after 1 year of age) display white-matter abnormalities, which are readily detected with magnetic resonance imaging, but these changes do not seem to be associated with any particular functional impairment. Structural brain changes have been reported in some patients, and epilepsy may be present. Moreover, patients have decreased peripheral nerve conduction velocity because of myelination defects. Severe heart failure is rare in MDC1A, but left ventricular dysfunction has been reported in about 30% of patients [78,79,82]. No treatment is currently available for this devastating disease.

### Mouse models for laminin α2 chain deficiency

**Figure 5:**
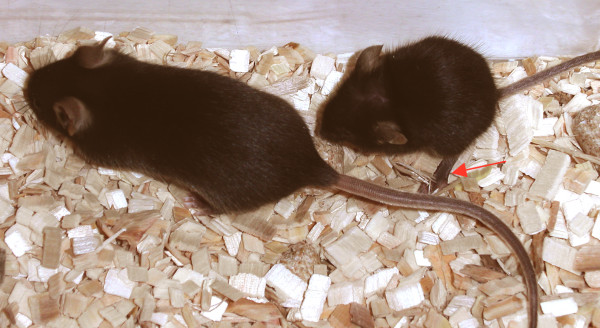
**A *dy***^***3K***^**/*dy***^***3K***^**laminin α2 chain-deficient mouse with a littermate (both 5 weeks old)**. Laminin α2 chain knockout mice have severe muscle wasting and growth retardation, resulting in dramatically decreased weight. Hind-limb paralysis (depicted with arrow), a result of peripheral neuropathy, is often seen before death.

A number of mouse models for laminin α2 chain deficiency exist, and in general they adequately model human disease. In addition, they confirm the relationship between laminin α2 chain expression and severity of disease [83] (Table 1 and references therein). The *dy/dy *mouse expresses reduced levels of an apparently normal laminin α2 chain, but the causative mutation remains to be identified. The *dy/dy *mouse displays moderate muscular dystrophy, peripheral neuropathy, heart fibrosis and defects in CNS myelination [11,12,84]. The *dy*^*2J*^/*dy*^*2J *^mouse harbors a mutation in the N-terminal (LN) domain, which leads to abnormal splicing and slightly reduced expression of a laminin α2 chain lacking this domain. These mice display a relatively mild muscular dystrophy and peripheral neuropathy [85,86]. Two further mouse models have been generated by homologous recombination: the *dy*^*W*^/*dy*^*W *^mouse expresses small amounts of a truncated α2 chain lacking the LN domain whereas the *dy*^*3K*^/*dy*^*3K *^mouse (Figure 5) is completely deficient in the laminin α2 chain. Both *dy*^*W*^/*dy*^*W *^and *dy*^*3K*^/*dy*^*3K *^mice develop severe muscular dystrophy and die within a few weeks of age. They also exhibit pronounced hind-leg lameness [42,43,83]. Nevertheless, it seems that the phenotype of the *dy*^*3K*^/*dy*^*3K *^mouse is more severe than that of the *dy*^*W*^/*dy*^*W *^mouse, and it could be that the residual laminin α2 chain expression in *dy*^*W*^/*dy*^*W *^muscle is beneficial. The most recently described mouse model is the *dy*^*nmj417*^/*dy*^*nmj417 *^mouse, in which a single point mutation in the LN domain leads to normal levels of a mutated laminin α2 chain and mild muscular dystrophy [87].

**Table 1:** Mouse models for laminin α2 chain deficiency.

Mouse model	Mutation/protein product	Phenotype	Ref
*dy/dy*	Unknown spontaneous mutation/reduced expression of seemingly normal α2 chain	Lethal within 6 months of age. Moderate muscular dystrophy; peripheral neuropathy; defective central nervous system myelination; hearing loss; aberrant thymocyte development	[11,12,84,121,122]

*dy*^*2J*^/*dy*^*2J*^	Spontaneous mutation in LN domain^a^/slightly reduced expression of truncated α2 chain devoid of LN domain	Normal lifespan. Mild muscular dystrophy; peripheral neuropathy	[85,86 ]

*dy*^*W*^/*dy*^*W*^	Knock- out/severely reduced expression of truncated α2 chain devoid of LN domain	Lethal at 10 to 15 weeks of age. Severe muscular dystrophy; peripheral neuropathy	[43,83]

*dy*^*3K*^/*dy*^*3K*^	Knock-out/complete deficiency	Lethal at 4 weeks of age. Severe muscular dystrophy; peripheral neuropathy; impaired spermatogenesis; defective odontoblast differentiation	[42,123-125]

*dy*^*nmj417*^/*dy*^*nmf417*^	N-ethyl-N-nitrosourea-induced point mutation in LN domain/normal levels	Normal lifespan. Mild muscular dystrophy; peripheral neuropathy	[87]

*dy*^*Pas*^/*dy*^*Pas *^(now extinct)	Spontaneous retrotransposal insertion/severe deficiency	Died at 13 weeks of age. Severe muscular dystrophy; peripheral neuropathy	[126]

^a^LN = laminin N-terminal domain.

Although it can be debated whether mice are reliable as preclinical models for human disease, analyses of the various laminin α2 chain-deficient mouse models have led to a significant improvement in our understanding of development of MDC1A. More importantly, they have been valuable tools for the development of novel therapeutic approaches for laminin α2 chain deficiency.

### Pathogenesis of MDC1A muscle

**Figure 6:**
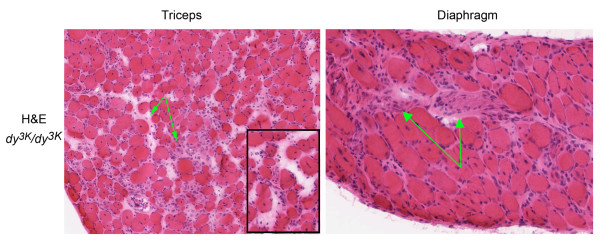
**Dystrophic features of laminin α2 chain muscle**. Hematoxylin and eosin staining of triceps and diaphragm cross-sections from a 3.5-week-old *dy*^*3K*^/*dy*^*3K *^mouse reveals fiber-size variability with predominance of small atrophic fibers. Regenerating fibers with centrally located nucleus and wide-spread fibrosis (green arrows) are also hallmarks of laminin α2 chain-deficient muscular dystrophy. Inset shows a higher magnification of *dy*^*3K*^/*dy*^*3K *^triceps.

Although the primary defect in MDC1A is known to be loss of the laminin α2 chain, the secondary molecular mechanisms ultimately leading to muscle degeneration have yet to be determined. Absence of laminin α2 from skeletal muscle gives rise to a marked dystrophic pattern with muscle fiber-size variation (with atrophy predominance), central nucleation and extensive fibrosis (Figure 6) [79]. Further features typical of MDC1A include disrupted basement membranes [11] and increased apoptosis [88,89]. It has been suggested that the laminin α2 chain confers a structural link (by binding to dystroglycan) from the ECM to the cytoskeleton, and that such linkage stabilizes the muscle-cell membrane and protects it from contraction-induced damage [90,91]. However, this hypothesis was challenged by Hall *et al*. [92]. Using a zebrafish laminin α2-chain mutant, they suggested that damage to the muscle fiber occurs by mechanically induced fiber detachment in the absence of sarcolemma rupture, and that detached fibers undergo apoptosis. Nevertheless, cell membranes are ruptured to some extent in animals with complete laminin α2 chain deficiency [70] and it has been proposed that laminin α2 chain binding to α-dystroglycan strengthens the sarcolemmal integrity [69]. The downstream signaling events leading to apoptosis remain to be deciphered, but recent data suggest that it includes glyceraldehyde-3-phosphate dehydrogenase (GAPDH)-Siah1-CBP/p300-p53 signaling [93]. However, there are relatively few apoptotic fibers in laminin α2 chain-deficient muscles [89,93], hence there must be other mechanisms underlying the muscle wasting seen in MDC1A. Both the ubiquitin-proteasome system and the autophagy-lysosome pathway play key roles in protein degradation in skeletal muscle cells [94]. Interestingly, we recently found that increased proteasomal degradation is a feature of *dy*^*3K*^/*dy*^*3K *^muscle [95], and preliminary unpublished data indicate that there might also be excessive autophagy in *dy*^*3K*^/*dy*^*3K *^muscle. Defects in both of these degradative systems have also been found in other muscular dystrophies. For example, Duchenne muscular dystrophy pathogenesis may involve proteasomal degradation of dystrophin and the DGC [96], and autophagy is impaired (but not increased as in MDC1A) in collagen VI-deficient muscular dystrophy [97].

At the molecular level, absence of the laminin α2 chain affects the expression and localization of several other laminin chains and cell-surface receptors. In particular, expression of the laminin β2 chain is severely reduced from the sarcolemmal basement membranes in laminin α2 chain-deficient muscle [44]. Conversely, laminin α4 (and α5 chain to some extent) is increased at this site [9,98]. However, it does not seem to compensate for the absence of the laminin α2 chain, presumably because the laminin α4 chain cannot bind α-dystroglycan [99], or possibly because that the laminin α4 chain is not upregulated in sufficient amounts. In extraocular muscles, which have a number of differences from other skeletal muscles, the laminin α4 chain is strongly expressed in the basement membrane adjoining the sarcolemma, and its expression is further enhanced in the extraocular muscle of *dy*^*3K*^/*dy*^*3K *^animals. Interestingly, laminin α2 chain-deficient extraocular muscles are spared from dystrophic changes, and it has been hypothesized that binding of the laminin α4 chain to integrin α7β1 may protect the extraocular muscles from damage [100,101].

Changes in the expression of laminin-211 receptors might also contribute to the pathology of MDC1A. A dramatic decrease of integrin α7 subunit in muscle from laminin α2 chain-deficient patients and mice [60,102,103], and a striking impairment of its deposition at the sarcolemma [104], has been noted, suggesting that integrin α7β1 signaling is abolished. By contrast, expression of β-dystroglycan at the sarcolemma, is upregulated in laminin α2 chain-deficient mouse muscle [104,105]. However, conflicting data have been reported on α-dystroglycan expression, with its production either not found to be significantly affected by laminin α2 chain deficiency [60,103] or shown to be moderately increased [104]. Moll *et al*. and Jimenez-Mallebrera *et al*. reported severe reduction of α-dystroglycan core protein [105,106]. The precise physiological outcomes of receptor alterations remain largely unknown, but altogether they point towards a central position of laminin-211 in regulating the expression of α7β1 and dystroglycan.

### Amelioration of disease in mice

**Figure 7:**
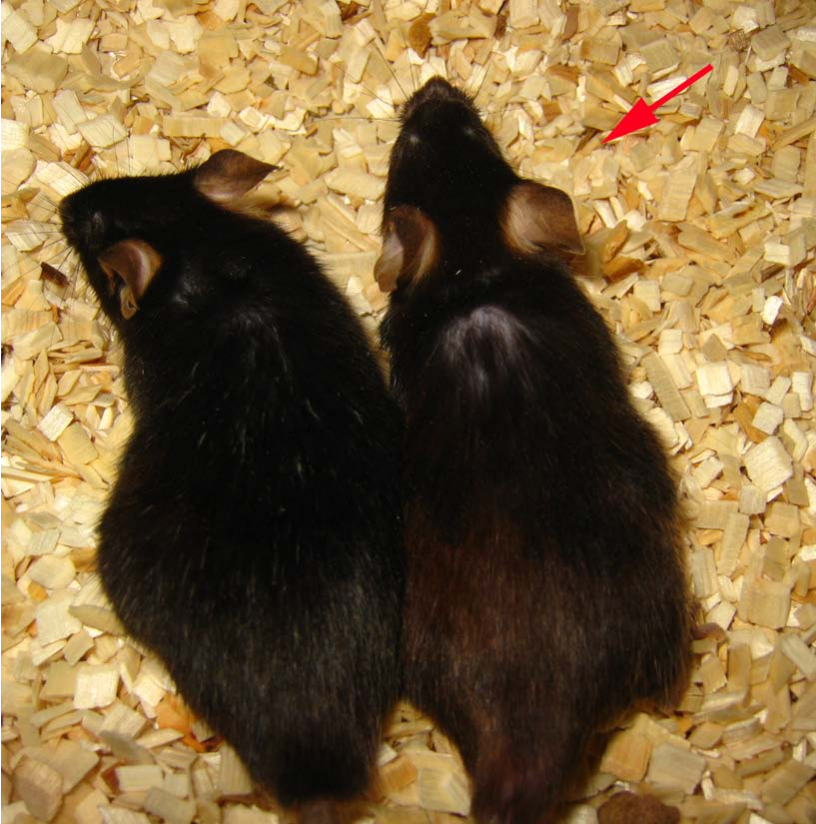
**A 2-year-old laminin α2 chain-deficient mouse (*dy*^*3K*^/*dy*^*3K*^) overexpressing laminin α1 chain, with a wild-type littermate**. Laminin α1 chain overexpression resulted in a remarkable improvement of overall health and prevention of muscular dystrophy that persisted throughout life.

Several strategies to combat disease in MDC1A mouse models have been explored during the past decade. Because the basement membrane is affected in MDC1A, many of these approaches have targeted the expression of ECM proteins. Transgenic expression of the laminin α1 and α2 chains, mini-agrin, and cytotoxic T cell GalNac transferase have been found to compensate for laminin α2 chain deficiency in mice [43,105,107-109]. *Dy*^*W*^/*dy*^*W *^mice bred to overexpress linker molecules (for example, mini-agrin, full-length agrin, agrin-perlecan fusion protein) between the laminin α4 chain and dystroglycan have a prolonged lifespan and significantly improved muscle tissue. Importantly, it was also found that mini-agrin can slow down the progression of disease at every stage [105,107,110]. The effects of laminin α1 chain overexpression in the neuromuscular system have also been extensively studied, and it has been shown that *dy*^*3K*^/*dy*^*3K *^mice overexpressing the laminin α1 chain have a near-normal lifespan and display considerably improved muscle, heart and nerve morphology and function [48,108,111] (Figure 7; see Additional file 1). The reduction in muscle fibrosis was particularly marked in these animals. Because transgenic expression of laminin α1 chain reconstituted integrin α7 at the sarcolemma [104], we reasoned that the laminin α1 chain mediated reduction of laminin α2 chain deficient muscular dystrophy mainly involves integrin α7β1 (which also binds laminin α1 chain with high affinity [112]). Consistent with this notion, a laminin α1 chain devoid of the dystroglycan binding site but retaining the integrin α7β1 binding domain significantly increased the lifespan of *dy*^*3K*^/*dy*^*3K *^mice and partially rescued dystrophic muscles, in particular the diaphragm [70]. However, subtle muscle defects (that is, residual degeneration) were seen in old animals overexpressing full-length laminin α1 chain [111]. Hence, it is possible that laminin α1 chain interactions with integrin α7β1 and dystroglycan might trigger different signaling cascades to those triggered by the laminin α2 chain.

Despite significant therapeutic benefits in mice, it is important to realize that these transgenic approaches are not clinically feasible. Therefore, adenoassociated virus-mediated gene transfer of mini-agrin was tested in *dy*^*W*^/*dy*^*W *^and *dy/dy *mice. Notably, systemic gene delivery of mini-agrin improved the overall phenotype and muscle function in treated animals [113].

Several approaches aimed at assuaging the secondary defects in MDC1A, instead of targeting the primary deficiency, have also been undertaken. As increased apoptosis had been suggested to contribute to the pathology of MDC1A, Miller *et al*. caused either inactivation of the proapoptotic protein Bax or overexpression of the antiapoptotic protein Bcl-2 in *dy*^*W*^/*dy*^*W *^animals [114,115]; both of these genetic interventions improved the health of the animals. Overexpression of Bcl-2 had no major effect in dystrophin-deficient mice, indicating that Bcl-2-mediated apoptosis is a more significant contributor to the pathogenesis of MDC1A than that of Duchenne muscular dystrophy [115]. The same group also recently explored the use of anti-apoptotic pharmacologic treatment. Interestingly, treatment with minocycline or doxycycline increased the lifespan of *dy*^*W*^/*dy*^*W *^animals and lessened muscle pathology [116]. Similarly, treatment with omigapil, which inhibits GAPDH-Siah1-mediated apoptosis, ameliorated the pathological features in *dy*^*W*^/*dy*^*W *^animals [93]. Recently, it was also established that mitochondria isolated from *dy*^*W*^/*dy*^*W *^muscle are swollen. This is a typical feature of abnormal opening of the permeability transition pore caused by a strong increase in intracellular calcium (which may be detrimental for the muscle cell). Persistent opening may cause mitochondrial rupture and subsequent cell death. Laminin α2 chain-deficient *dy*^*W*^/*dy*^*W *^mice devoid of cyclophilin-D, which is a regulatory protein of the permeability transition pore, displayed reduced muscular dystrophy pathology [117]. Additionally, because enhanced proteasomal degradation is a feature of laminin α2 chain-deficient muscle [95], we hypothesized that inhibition of the proteasome would lessen the myopathology, and indeed, treatment with the proteasome inhibitor MG-132 significantly improved the lifespan and muscle morphology of *dy*^*3K*^/*dy*^*3K *^mice [95].

Finally, cell therapy has been evaluated in mouse models of MDC1A. Myoblast and CD90-positive cell transplantation led to laminin α2 chain expression in *dy/dy *and *dy*^*3K*^/*dy*^*3K *^mice, respectively, but no further improvement in the animals, was reported [118,119]. However, bone-marrow transplantation improved life span, growth rate, muscle strength and importantly, respiratory function of *dy/dy *animals [120].

Altogether, considering that laminin α2 chain deficiency seems to affect different cellular events, combinatorial treatment strategies (for example, apoptosis and proteasome inhibitors together with replacement therapy) may be relevant for MDC1A. Moreover, bearing in mind that MDC1A is associated with peripheral neuropathy, therapies that also alleviate the neurologic dysfunction should be favored. Previous studies found that motor nerve pathology could not be prevented by muscle-specific expression of laminin α2 chain [43] and mini-agrin [105], whereas ubiquitous expression of the laminin α1 chain significantly reduced peripheral neuropathy [48]. In addition, inactivation of Bax s [114] and treatment with doxycycline [116] were reported to be beneficial for the condition of motor neuron.

## Conclusion

A great deal is known about the structure and function of laminin-211, and advances concerning the development of future therapies have been made for murine laminin α2 chain-deficient muscular dystrophy. Absence of the laminin α2 chain does not only affect skeletal muscle but also several non-muscle tissues. Analysis of these organs has been hampered by the relatively early death of the animals. It would therefore be informative to analyze the non-muscle organs in animals that have been rescued from the muscle defects or to generate mice with a tissue-specific disruption of the laminin α2 chain. Furthermore, the targeted genetic elimination of individual laminin domains (in particular the LG domains) would be valuable to understand their role *in vivo*. Finally, elucidation of laminin α2 chain-induced signal transduction pathways is an important task. Such studies would be helpful to further clarify the details of laminin α2 chain function to design future treatment for MDC1A.

## Abbreviations

CBP: cAMP response element binding protein (CREB) binding protein.

## Competing interests

The authors declare that they have no competing interests.

## Authors' contributions

KIG And MD wrote the paper.

## Authors' information

KIG is a post-doctoral student at the Department of Experimental Medical Science, University of Lund, with a PhD in cell and molecular biology, specializing in preclinical studies of laminins and muscle disease. MD is a professor in muscle biology at the Department of Experimental Medical Science, University of Lund, with a PhD in animal physiology, specializing in preclinical studies of laminins and muscle disease.

## Supplementary Material

Additional file 1 **Supplemental video 1. A 2-year-old laminin α2 chain-deficient mouse (*dy***^***3K***^**/*dy***^***3K***^**) overexpressing laminin α1 chain together with a wild-type littermate**. The rescue mouse is denoted with a blue pointer at the beginning of the f. Both mice were placed in a new cage. The *dy*^*3K*^LMα1 mouse is as active as wild-type littermate; it explores the cage and often stands up on its hind limbs. Click here for file
